# Wernicke Encephalopathy as a Rare Complication of Antituberculosis Treatment: A Case Report

**DOI:** 10.7759/cureus.110095

**Published:** 2026-06-02

**Authors:** Khadija Chaanoun, Bouchra Dassouli, Wissal Bouddine, Hanane Benjelloun, Nahid Zaghba

**Affiliations:** 1 Department of Pulmonology, Ibn Rochd University Hospital, Casablanca, MAR

**Keywords:** case report, corticosteroids, hepatotoxicity, isoniazid, mri, self-medication, thiamine or vitamin b1 deficiency, tuberculosis, vomiting, wernicke encephalopathy

## Abstract

Wernicke encephalopathy (WE) is an acute neurological emergency caused by severe thiamine deficiency. Although classically associated with chronic alcoholism, it may also occur in nonalcoholic settings, including during prolonged antituberculosis treatment. We report the case of a 29-year-old woman treated for bacteriologically confirmed pulmonary tuberculosis who developed recurrent episodes of severe drug-induced hepatotoxicity, requiring treatment interruption and modification. Despite the introduction of an alternative antituberculosis regimen, she experienced persistent vomiting followed by the acute onset of cerebellar ataxia and ophthalmoplegia. Brain MRI revealed bilateral and symmetric fluid-attenuated inversion recovery hyperintensities of the medial thalami, the periaqueductal gray matter, and the mammillary bodies, consistent with WE. Plasma thiamine level was markedly reduced (55 nmol/L). High-dose IV thiamine (500 mg three times daily for five days), followed by oral supplementation, led to rapid and complete neurological recovery. WE is a rare but potentially severe complication in patients receiving antituberculosis therapy, particularly in the presence of persistent vomiting and hepatic toxicity. Early recognition and prompt treatment with thiamine are essential to prevent irreversible neurological sequelae. Preventive thiamine supplementation should be considered in high-risk patients.

## Introduction

Wernicke encephalopathy (WE) is an acute neuropsychiatric syndrome, potentially fatal, resulting from a deficiency of thiamine (vitamin B1). Thiamine acts as an essential cofactor for key enzymes in cerebral oxidative metabolism, including pyruvate dehydrogenase and alpha-ketoglutarate dehydrogenase. Its deficiency leads to impaired neuronal energy production and selective injury to metabolically vulnerable regions, particularly the medial thalami, mammillary bodies, and periaqueductal gray matter, ultimately producing the clinical syndrome of WE. Body thiamine stores are limited, typically lasting only two to three weeks in the absence of adequate intake. Its prevalence in autopsy studies is estimated to be between 0.4% and 2.8%, suggesting substantial underdiagnosis in clinical practice [1,2]. The classic triad (oculomotor abnormalities such as ophthalmoplegia or nystagmus, cerebellar ataxia, and altered consciousness) is complete in only 10-33% of patients according to published series [3,4]. Untreated, WE is associated with substantial mortality, and a significant proportion of survivors progress to chronic Korsakoff syndrome [5].

Although classically associated with chronic alcoholism, WE is increasingly recognized in nonalcoholic settings: hyperemesis gravidarum, bariatric surgery, malabsorption, malnutrition, malignancies, prolonged parenteral nutrition, eating disorders, and chronic infections such as tuberculosis [4,6-8].

Antitubercular treatment represents a particularly at-risk context due to three converging mechanisms: drug-induced hepatotoxicity impairing hepatic thiamine storage and activation, prolonged digestive intolerance reducing oral thiamine intake, and the interference of isoniazid with B-vitamin metabolism. However, observations of WE occurring during antitubercular treatment remain scarcely reported in the literature [9]. We report this case to alert clinicians to this underrecognized association, whose early recognition determines the neurological prognosis.

## Case presentation

A 29-year-old woman, with no significant medical history and no alcohol or tobacco use, was admitted for reintroduction of antitubercular treatment following a previous episode of drug-induced hepatotoxicity. Her history revealed self-medication with prednisone 5 mg/day for six months, used for nonmedical purposes (weight gain), spontaneously discontinued by the patient a few weeks prior to the diagnosis of pulmonary tuberculosis. On admission, her BMI was 19.5 kg/m² (weight 59 kg, height 174 cm), within normal limits. Vital signs were as follows: blood pressure 120/60 mmHg, heart rate 85 bpm, temperature 37.5°C, and oxygen saturation 96% on room air. HIV serology was negative, and the pregnancy test (beta-human chorionic gonadotropin) was also negative. Morning cortisol level was within normal limits, excluding residual adrenal insufficiency from prolonged corticosteroid exposure.

The initial diagnosis of pulmonary tuberculosis had been bacteriologically confirmed, and a first-line antitubercular regimen (fixed-dose combination ERIP-K4, four tablets daily, weight-adjusted) had been initiated. No preventive vitamin supplementation, including thiamine or pyridoxine, had been prescribed at the time of treatment initiation.

The initial chest radiograph showed confluent heterogeneous infiltrative opacities of low density, with poorly defined borders and multiple cavitary areas, involving the middle lobes (right middle lobe and lingula) and lower lobes bilaterally, with involvement of the upper segments (Fowler’s segments). The diagnosis was bacteriologically confirmed (Figure 1).

**Figure 1:**
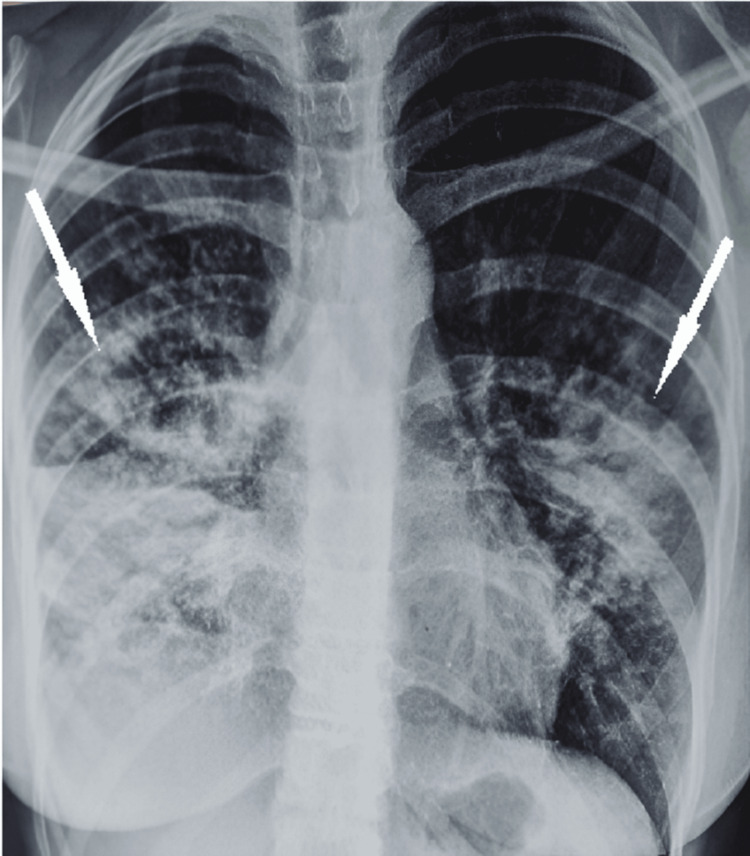
Frontal chest radiograph before treatment Arrows indicate confluent heterogeneous infiltrative opacities of low density, with poorly defined borders and multiple cavitary areas, involving the middle lobes (right middle lobe and lingula) and lower lobes bilaterally, with involvement of the upper segments (Fowler’s segments).

After two weeks of treatment (approximately Day 14), the patient developed persistent vomiting, myalgia, abdominal pain, and marked asthenia. Liver function tests revealed significant hepatocellular injury, with aspartate aminotransferase 198 U/L, alanine aminotransferase 227 U/L, total bilirubin 70 µmol/L (conjugated 26 µmol/L), and a markedly decreased prothrombin time of 25%, prompting immediate interruption of antitubercular treatment. After normalization of hepatic parameters (approximately Day 28), progressive reintroduction of first-line antituberculars was attempted in the following sequence: ethambutol was reintroduced first and was hepatically well tolerated; isoniazid was subsequently reintroduced with satisfactory hepatic tolerance. However, reintroduction of rifampicin triggered a recurrence of hepatotoxicity, leading to its definitive discontinuation, while pyrazinamide, recognized as the most hepatotoxic agent among first-line antituberculars, was not reintroduced. An alternative regimen combining IV amikacin (500 mg twice daily), levofloxacin (500 mg daily), ethambutol (800 mg daily), and isoniazid with progressive escalation up to 300 mg daily was therefore instituted (approximately Day 35), with satisfactory hepatic tolerability. Pyridoxine (vitamin B6) supplementation at 50 mg/day was then introduced, given the resumption of isoniazid and the at-risk nutritional context; however, no thiamine supplementation was initiated at that stage.

Despite good hepatic tolerance of the alternative regimen, the patient continued to experience persistent vomiting, with approximately five episodes per day for one week and markedly reduced oral intake over a total period exceeding three weeks. Given the persistence of vomiting despite the change of antitubercular regimen, a psychiatric evaluation was performed to rule out an eating disorder. No diagnosis of anorexia nervosa, bulimia nervosa, or any other eating disorder was retained. Parenteral nutrition (Oliclinomel) was initiated two days before the onset of neurological symptoms, without concomitant thiamine supplementation, a recognized precipitating factor for WE in thiamine-depleted patients.

Seven days after initiation of this regimen, she developed acute neurological symptoms. Neurological examination at onset revealed cerebellar ataxia with a wide-based, unstable gait and inability to perform tandem walking. Cerebellar dysmetria was present on finger-nose testing bilaterally. Oculomotor examination disclosed multidirectional rotatory and horizontal nystagmus on smooth pursuit and lateral gaze, along with a right sixth cranial nerve (abducens) palsy, without complete ophthalmoplegia. The remaining cranial nerves (III, IV, V, and VII-XII) were intact. Motor strength was preserved in all four limbs, deep tendon reflexes were normoactive, and sensory examination, including light touch, pinprick, and vibration sense, was normal, effectively excluding an associated isoniazid-induced peripheral neuropathy. The patient remained fully oriented to time, place, and person, with no confabulation, no anterograde memory deficit on clinical interview, and no language disturbance. Formal neuropsychological testing was not performed at this stage, which represents an acknowledged limitation in the characterization of cognitive status.

Laboratory workup at neurological onset showed sodium 130 mmol/L, potassium 4.3 mmol/L, normal fasting glucose, normal renal function, and a normal complete blood count without macrocytic anemia, actively excluding B12 and folate deficiencies. Examination found no asterixis, and the liver function tests had normalized for cytolysis markers and hepatic synthetic function (prothrombin time recovered to over 70%), effectively excluding hepatic encephalopathy. It should be noted that serum magnesium and phosphate levels were not measured at the time of neurological presentation, which represents a limitation: hypomagnesemia can impair cellular thiamine uptake, and hypophosphatemia may complicate refeeding in thiamine-deficient patients, both potentially influencing the response to supplementation.

Brain MRI revealed bilateral and symmetric fluid-attenuated inversion recovery (FLAIR) hyperintensities of the medial thalami bordering the third ventricle, as well as symmetric hyperintensity of the mammillary bodies, with corresponding diffusion-weighted imaging hyperintensity and elevated apparent diffusion coefficient values reflecting vasogenic edema, consistent with WE (Figure 2).

**Figure 2:**
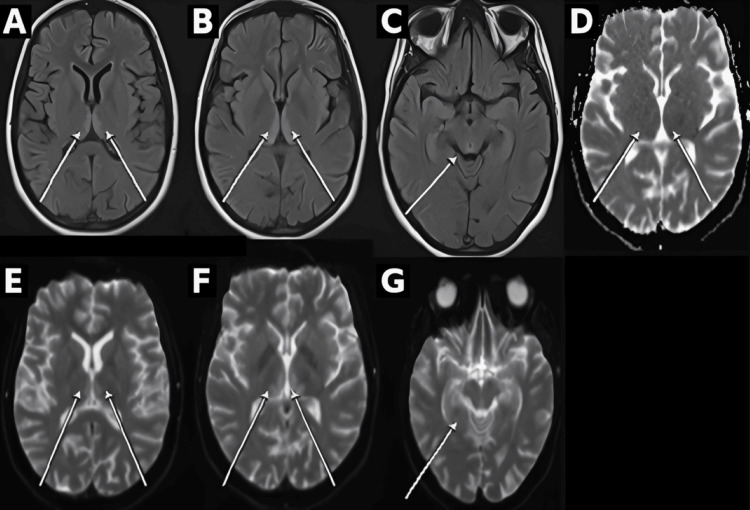
Axial brain MRI (A-C) Axial FLAIR sequences. (D-G) ADC maps derived from DWI. Panels A and B demonstrate symmetric bilateral FLAIR hyperintensity of the medial thalami (arrows). Panel C shows bilateral FLAIR hyperintensity of both mammillary bodies (arrow), highly characteristic of WE. Panels D, E, and F show elevated ADC values at the thalamic level (arrows), consistent with vasogenic edema. Panel G shows elevated ADC values at the level of the mammillary bodies (arrow), confirming vasogenic edema. ADC, apparent diffusion coefficient; DWI, diffusion-weighted imaging; FLAIR, fluid-attenuated inversion recovery; WE, Wernicke encephalopathy

Plasma thiamine concentration was decreased at 55 nmol/L (reference range: 67-200 nmol/L). Whole-blood thiamine measurement, which assesses the active thiamine diphosphate form stored in erythrocytes and is considered the gold standard for evaluating thiamine status, was not available at our institution. High-dose IV thiamine (500 mg three times daily for five days) was promptly initiated, followed by oral supplementation.

Neurological recovery followed a timeline consistent with published series: nystagmus showed near-complete resolution by Day 2, dysmetria and gait ataxia partially improved by Day 3, and tandem walking was restored by Day 4, with complete clinical resolution. At three-month follow-up, cranial nerve examination was normal, gait was stable with no residual ataxia, and no cerebellar signs were elicitable. Bedside cognitive assessment at three months demonstrated intact orientation, preserved immediate memory, and normal language function. No confabulation or signs suggestive of Korsakoff syndrome were elicited. Although no formal neuropsychological battery (Mini-Mental State Examination or Montreal Cognitive Assessment) was administered, the clinical picture was consistent with complete cognitive recovery. The absence of formal standardized testing represents a limitation that precludes definitive conclusions regarding subtle cognitive domains.

The follow-up chest radiograph showed marked regression of the previously described opacities, with improved bilateral parenchymal aeration, reflecting a favorable response to the adapted antitubercular regimen (Figure 3).

**Figure 3:**
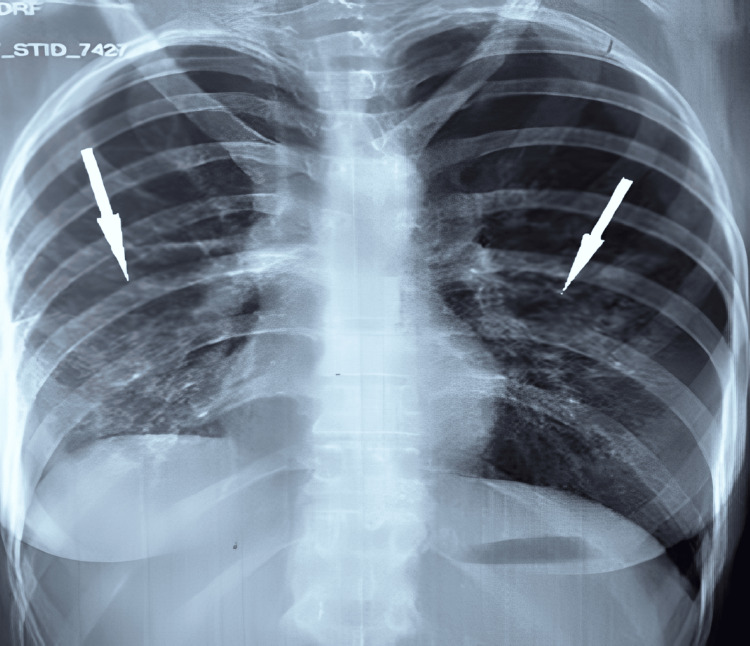
Frontal chest radiograph at three-month follow-up, on second-line antitubercular treatment and thiamine supplementation Arrows indicate marked regression of the previously described infiltrative opacities of the middle and lower lobes and clear improvement of bilateral parenchymal aeration, with persistence of discrete fibrotic sequelae, reflecting a good therapeutic response.

## Discussion

WE is an infrequently reported but potentially serious complication of prolonged antitubercular treatment. Although classically associated with alcoholism, WE may occur in nonalcoholic settings in the presence of persistent vomiting, hepatotoxicity, nutritional deficiencies, and absence of preventive thiamine supplementation [1,2,7]. A large multicenter study of 468 patients confirmed that nonalcoholic etiologies now represent a substantial proportion of cases, with diagnostic delays disproportionately affecting this subgroup [3].

The radiological presentation of our patient, confluent infiltrative opacities of the middle and lower lobes with involvement of the upper segments (Fowler’s segments), diverges from the classic pattern of adult post-primary tuberculosis, which classically predominates in the apical and posterior segments of the upper lobes in the majority of cases [10]. This so-called “atypical” distribution is best explained, in our case, by the antecedent of iatrogenic immunosuppression: prolonged corticosteroid use, including at doses below 15 mg/day of prednisone or equivalent, is associated with an increased risk of tuberculosis, with an adjusted odds ratio of approximately 2.8 according to the case-control study by Jick et al. [11]. The combination of a BMI in the lower normal range (19.5 kg/m²), absence of preventive vitamin supplementation, recurrent hepatotoxicity, and prolonged vomiting created a context particularly conducive to rapid thiamine depletion, since body stores typically last less than three weeks in the absence of intake [5].

Several differential diagnoses had to be systematically ruled out: hepatic encephalopathy was excluded by the normalization of liver function at the time of neurological symptoms, the absence of asterixis, and an MRI lesion distribution inconsistent with this diagnosis; toxic encephalopathy from antituberculars, particularly isoniazid or fluoroquinolones, classically presents with seizures or peripheral polyneuropathy rather than focal mesencephalic-diencephalic involvement [12]; deep cerebral venous thrombosis was excluded by the absence of venous abnormality on MRI; infectious encephalitis was deemed unlikely given the absence of fever, meningeal syndrome, and the symmetric and characteristic radiological distribution. Posterior reversible encephalopathy syndrome was ruled out by the absence of hypertension and an MRI pattern inconsistent with posterior cortical/subcortical involvement. Osmotic demyelination syndrome was considered, given the mild hyponatremia (Na 130 mmol/L) but excluded, as no rapid osmolar correction had been performed. Refeeding syndrome was considered, but the clinical and radiological picture was consistent with WE. Vitamin B12 and folate deficiencies were actively excluded by the absence of macrocytic anemia. The rapid reversibility of symptoms with IV thiamine constituted a major additional diagnostic argument in favor of WE.

Our patient met three of the four operational criteria of Caine et al. (nutritional deficit secondary to prolonged vomiting, oculomotor abnormalities consisting of nystagmus and abducens palsy, and cerebellar dysfunction with ataxia and dysmetria), well above the threshold of two of four criteria, which had a sensitivity of 85% for WE in autopsied alcoholic patients in the original validation cohort [13]. Brain MRI plays a central role in the diagnostic evaluation: typical abnormalities include bilateral and symmetric FLAIR hyperintensities involving the medial thalami (~80% of cases) and the periaqueductal gray matter (~59%), with more variable involvement of the mammillary bodies (~45%) and the tectal plate (~36%) [2,8,14]. In our patient, MRI demonstrated the two most frequent signs, symmetric hyperintensity of the medial thalami and periaqueductal gray matter, with corresponding diffusion restriction in the acute phase reflecting intramyelinic/cytotoxic edema [15]. High-dose IV thiamine should be initiated immediately upon clinical suspicion, without waiting for thiamine assay results or MRI confirmation.

The pathophysiology of WE during antitubercular treatment is multifactorial. Antitubercular hepatotoxicity, reported in 5-28% of patients receiving isoniazid, rifampicin, or pyrazinamide, frequently causes prolonged vomiting and reduced oral intake [16]. Furthermore, isoniazid interferes with the metabolism of water-soluble B vitamins. Although no direct biochemical interaction between isoniazid and thiamine is clearly established, the combination of reduced oral intake secondary to vomiting and hepatocellular injury, altering thiamine storage and activation, provides a coherent pathophysiological basis for the depletion observed in our patient [12,17].

Management relies on urgent administration of high-dose IV thiamine, followed by oral supplementation [1,18,19]. The recommendations of the European Federation of Neurological Societies advocate a minimum dose of 200 mg three times daily, and recent data support 500 mg three times daily for five days in severe forms [5,16,20]. Administration of glucose-containing fluids before thiamine substitution may precipitate or worsen WE and should be avoided [16].

Beyond the clinical aspects of management, this case highlights two public health issues. First, self-medication with corticosteroids for nonmedical indications exposes patients to an increased risk of opportunistic infections, including tuberculosis, and calls for stronger pharmaceutical regulation and patient education. Second, the absence of a standardized protocol for preventive thiamine supplementation in at-risk tuberculosis patients remains a notable gap: while pyridoxine is widely recommended with isoniazid, thiamine is not systematically administered despite a real risk in patients presenting with precarious nutritional status, digestive intolerance, or hepatotoxicity.

## Conclusions

WE should be considered in patients on antitubercular treatment who develop persistent digestive symptoms or acute neurological signs, even in the absence of confusion or a history of alcohol use disorder. Early recognition and rapid administration of high-dose thiamine are essential to prevent irreversible sequelae. In our patient, the rapid initiation of IV thiamine therapy resulted in complete clinical neurological recovery by Day 4, including resolution of oculomotor, cerebellar, and gait abnormalities, with no signs of Korsakoff syndrome on bedside cognitive assessment, although formal neuropsychological testing was not performed. No residual deficits were noted at three months. Conversely, diagnostic delays may expose patients to irreversible Korsakoff syndrome or even death; thiamine administration should therefore never be withheld pending confirmatory investigations.

This observation also raises a public health concern regarding self-medication with corticosteroids, a known yet frequently overlooked risk factor for tuberculosis. Overall, this case supports consideration of preventive thiamine supplementation in tuberculosis patients presenting with identifiable risk factors for thiamine depletion, prolonged vomiting, hepatotoxicity due to rifampicin or pyrazinamide, or precarious nutritional status, in line with existing expert recommendations. Given the limitations inherent to a single case report, prospective studies are needed to define formal preventive supplementation protocols in this population.
